# Competition between recombination and extraction of free charges determines the fill factor of organic solar cells

**DOI:** 10.1038/ncomms8083

**Published:** 2015-05-07

**Authors:** Davide Bartesaghi, Irene del Carmen Pérez, Juliane Kniepert, Steffen Roland, Mathieu Turbiez, Dieter Neher, L. Jan Anton Koster

**Affiliations:** 1 Department of Photophysics and Optoelectronics, Zernike Institute for Advanced Materials, University of Groningen, Nijenborgh 4, NL-9747AG Groningen, The Netherlands; 2 Dutch Polymer Institute, P. O. Box 902, 5600AX Eindhoven, The Netherlands; 3 Institute of Physics and Astronomy, University of Potsdam, Karl-Liebknecht-Strasse 24-25, 14476 Potsdam, Germany; 4 BASF Schweiz AG, Schwarzwaldallee 215, CH-4002 Basel, Switzerland

## Abstract

Among the parameters that characterize a solar cell and define its power-conversion efficiency, the fill factor is the least well understood, making targeted improvements difficult. Here we quantify the competition between charge extraction and recombination by using a single parameter *θ*, and we demonstrate that this parameter is directly related to the fill factor of many different bulk-heterojunction solar cells. Our finding is supported by experimental measurements on 15 different donor:acceptor combinations, as well as by drift-diffusion simulations of organic solar cells in which charge-carrier mobilities, recombination rate, light intensity, energy levels and active-layer thickness are all varied over wide ranges to reproduce typical experimental conditions. The results unify the fill factors of several very different donor:acceptor combinations and give insight into why fill factors change so much with thickness, light intensity and materials properties. To achieve fill factors larger than 0.8 requires further improvements in charge transport while reducing recombination.

## Abstract (web summary)

The open circuit voltage, short circuit current and fill factor determine the conversion efficiency of organic solar cells, yet the fill factor is not well understood. Here, Bartesaghi *et al*. demonstrate the fill-factor dependence on the ratio of the recombination and extraction rates of free charges.

## Graphical abstract

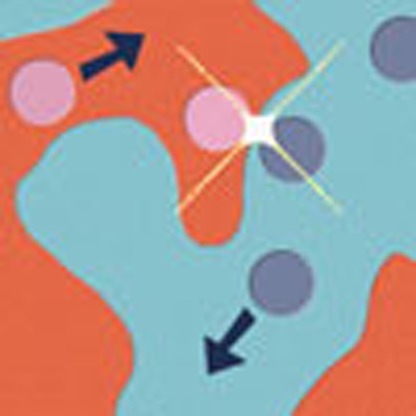

Organic photovoltaic devices (OPVs) represent a highly attractive choice for harnessing solar energy in terms of low cost, easiness of production, flexibility and environmental sustainability. Researchers have therefore put considerable effort in the development of this technology, seeking to increase both the efficiency and the stability of OPV devices^1^,^2^,^3^,^4^. The achievement of these goals is strongly dependent on the understanding of the physics of the devices.

To characterize a solar cell, three parameters are usually considered: the open circuit voltage (*V*_oc_), the short circuit current (*J*_sc_) and the fill factor (FF), defined as


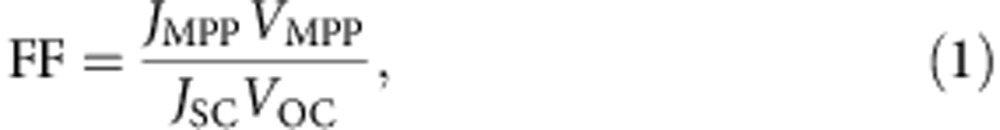


where *J*_MPP_ and *V*_MPP_ are the current density and voltage at the maximum power point (MPP), respectively (Fig. 1).

Over recent years, a good understanding of the physical phenomena governing *J*_sc_^5^,^6^ and *V*_oc_^7^,^8^,^9^ has been achieved, while a similar understanding is lacking for the FF. If the generation of charges changes significantly between open-circuit and short-circuit conditions, then this will influence the FF. Such field-dependent generation of charge carriers has been shown to be the main determinant in some donor/acceptor combinations^10^,^11^. In most of the high-efficiency OPV systems, geminate recombination is greatly reduced and bimolecular recombination is the main mechanism for charge recombination^12^. It is then the competition between recombination and extraction of charges that principally determines the dependence of the photocurrent on bias, and hence the FF^13^. Some authors combined transport and recombination by treating the problem in terms of the mobility-lifetime product^14^,^15^.

Whether the recombination is dominant over the extraction or vice versa depends on a complex interplay between the effects of thickness^14^,^16^, charge transport^17^,^18^, recombination strength^13^,^19^ and light intensity^20^. Although many publications in the literature relate the differences in FF between different solar cells to one parameter, such as the thickness^14^,^16^ or the light intensity^20^, it has been recently shown that one has to consider all the dynamic processes in the device^21^ to understand changes in FF. A complete and systematic understanding of the interplay of all the factors mentioned above, which may translate into significant improvements of the FF and hence of the power-conversion efficiency, is still lacking.

In this study we focus on the FFs of a large number of solar cells, using both simulations by means of drift-diffusion modelling^22^ and experimental data. The simulations are performed by varying the charge-carrier mobilities, recombination rate, light intensity, energy levels and active-layer thickness over a wide range, to reproduce typical experimental conditions. Experimental data are collected from a wide variety of donor:acceptor combinations, including systems that exhibit a weak field dependency in the photogeneration of charges. Both simulated and experimental devices have ohmic contacts, as it is the case for the most efficient organic solar cells. For each combination, we measure charge-carrier mobilities and bimolecular recombination rates using a combination of steady-state and transient techniques, and we use these quantities to estimate the recombination and extraction times. In addition, we include experimental data taken from the literature, for devices based on polymer:fullerene^16^,^23^ and small-molecule^17^ systems. We introduce a dimensionless parameter *θ* that quantifies the ratio of the recombination and extraction rates. This single parameter unifies the effects of charge-carrier mobilities, recombination rate, light intensity and thickness. When all the FFs of the solar cells studied are plotted versus *θ*, the data collapse onto one universal curve, showing that the parameter *θ* is suitable to quantify the competition between recombination and extraction of free charges. Our results unify the FFs of 15 very different donor:acceptor combinations and help explain why FFs change so much with thickness, light intensity and materials properties, which opens up the possibility of targeted improvement. We show that to achieve FFs of over 0.8, further improvements in charge transport are needed while reducing recombination.

## Results

### Drift-diffusion model and material parameters

Organic solar cells are generally based on the combination of two materials, one electron donor and one electron acceptor. This bulk heterojunction (BHJ) can be considered as one effective semiconductor medium, whose valence and conduction bands are represented by the higher occupied molecular orbital of the donor (HOMO_D_) and by the lowest unoccupied molecular orbital of the acceptor (LUMO_A_), respectively. This effective medium is sandwiched between two metal electrodes. The charges photogenerated inside the active layer on absorption of photons move driven by the external electric field (drift) and by their concentration gradient (diffusion). The drift and diffusion of charges can be described by a model, presented in detail in ref. 22, which includes generation and recombination of free charge carriers, as well as the effect that the possible build-up of space charge has on the electric field. The generation of free charges is assumed to be field-independent. In spite of its simplicity, the monodimensional drift-diffusion model is a powerful tool to treat BHJ systems and it has been used by many groups in the past to properly describe the *J–V* curves of real devices^24^,^25^,^26^.

We perform monodimensional drift-diffusion modelling of the *J–V* curves of organic solar cells by using a set of parameters that are representative of a vast class of BHJs in different operating conditions. The ranges used for these parameters are listed in Table 1. As the light-absorbing materials used in OPV are typically strongly absorbing, the active layers can be relatively thin. A range of 60–260 nm is chosen in the simulations. The difference between LUMO_A_ and HOMO_D_ varies somewhat between solar cell materials; hence, this parameter is also varied. The contacts are assumed to be ohmic, that is, there are no energy barriers for the injection and extraction of electrons and holes at the cathode and anode, respectively. In the simulations, the carriers at the contact are assumed to be in thermal equilibrium with the electrodes^22^, which implies infinitely strong surface recombination of the minority micarriers. Using near-zero recombination velocities (10^−4^ m s^−1^) for the electrons (holes) near the anode (cathode) yields virtually the same results for FF versus *θ* (see Supplementary Note 1 and Supplementary Fig. 1a). We also verified that our results are not influenced by the exact carrier densities at the contacts (see Supplementary Note 1 and Supplementary Fig. 1b).

The mobilities of electrons and holes characterize the charge transport. For simplicity, we assume the mobilities to be not dependent on the electric field and on the charge density. Our simulations consider bimolecular recombination as the main loss mechanism of free charges. The strength of bimolecular recombination is given by


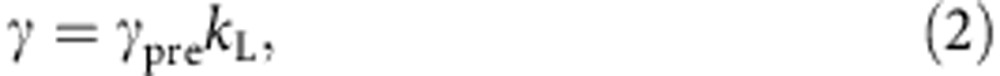


where *γ*_pre_ is a dimensionless reduction prefactor and *k*_L_ is the classical Langevin recombination coefficient^27^


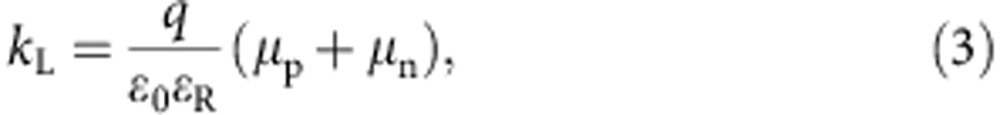


in which *ɛ*_0_ is the vacuum dielectric constant, *ɛ*_R_ is the relative dielectric constant of the blend, *q* is the elementary charge, and *μ*_p_ and *μ*_n_ are the hole and electron mobilities, respectively (see Table 1). As there is no experimental evidence of organic solar cells with a recombination strength higher than what follows from equation (3), we consider the Langevin expression as an upper limit for the bimolecular recombination strength. The reduction of the classical Langevin recombination rate in BHJs has been observed experimentally in some polymer:fullerene^28^, polymer:polymer^29^,^30^ and small-molecule solar cells^31^; typical values for the reduction prefactor *γ*_pre_ are between 1 and 1 × 10^−3^.

The generation rate of free charges *G* is proportional to light intensity; we vary *G* to simulate solar cells under different illumination conditions, from 10^25^ to 10^28^ m^−3^ s^−1^, roughly corresponding to 10^−3^–1 sun intensity.

### Donor and acceptor materials

**Figure 2:**
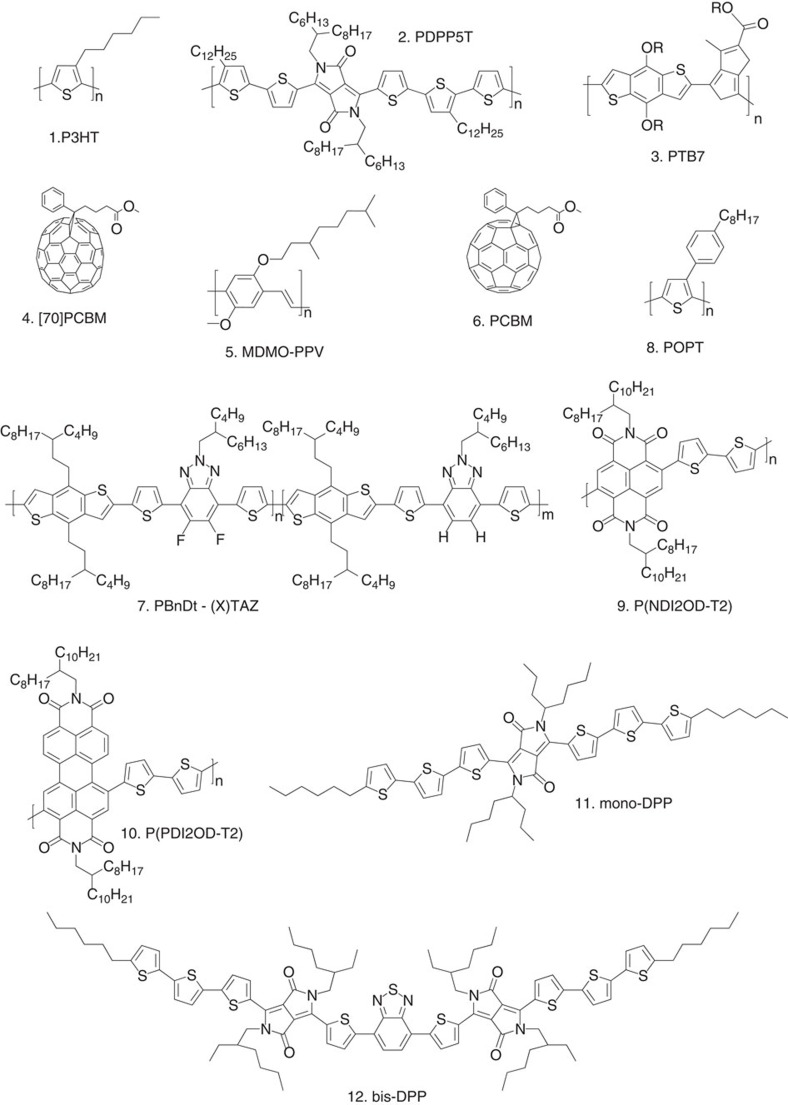
Chemical structures of the materials. Chemical structures of the materials considered in this study.

Experimental data from BHJ solar cells are based on the combination of a variety of donor and acceptor materials, fabricated in different laboratories and characterized by different techniques (see Methods section). To avoid losses due to the series resistance of the contacts^32^, devices with small active area (0.01–0.04 cm^2^) are fabricated. To draw conclusions that are valid for a large class of OPVs, three families of BHJs are considered: polymer:fullerene, polymer:polymer and small-molecule solar cells. The chemical structures of the materials are shown in Fig. 2.

#### Polymer:fullerene solar cells

The polymers used in this study are poly(3-hexylthiophene) (P3HT)^33^, diketopyrrolopyrrole-quinquethiophene alternating copolymer (PDPP5T)^34^ and polythieno[3,4-b]-thiophene-co-benzodithiophene (PTB7)^2^. These polymers are mixed with [6,6]-phenyl-C_71_-butyric acid methyl esther ([70]PCBM). Devices based on these polymer:fullerene systems are fabricated and characterized following the procedures described in the Methods section.

In addition, we include experimental data from the literature. These include a well-known system, poly(2-methoxy-5-(3′,7′-dimethyl octyloxy)-*p*-phenylene vinylene) (MDMO-PPV) blended with [6,6]-phenyl-C_61_-butyric acid methyl esther PCBM^16^, and a more recent series of donor–acceptor copolymers in which the electron donor benzodithiophene (BnDT) is alternated with the electron acceptor 2-alkyl-benzo[d][1,2,3]triazole or its fluorinated analogue and blended with PCBM. The data are taken from a recent paper, in which Li *et al*.^23^ compared the performance of BnDT-(X)TAZ:PCBM systems with different content of fluorinated groups.

#### Polymer:polymer solar cells

We consider the mixture of the donor polymers P3HT and poly[3-(4-octylphenyl)thiophene] (POPT)^35^,^36^ with the acceptor poly([N,N′-bis(2-octyldodecyl)-naphtalene-1,4,5,8-bis(dicarboximide)-2,6-diyl]-alt-5,5′-(2,2′-bithiophene)) (P(NDI2OD-T2)), which is also referred to as N2200 (ref. 37) and its perylene-based analogue (P(PDI2OD-T2))^38^.

#### Small-molecule solar cells

We include data from the literature regarding 2,5-di-(2-ethylhexyl)-3,6-bis-(5′′-n-hexyl-[2,2′,5′,2′′]terthiophen-5-yl)-pyrrolo[3,4-c]pyrrole-1,4-dione (mono-DPP) and 4,7-bis{2-[2,5-bis(2-ethylhexyl)-3-(5-hexyl-2,2′:5′,2′′-terthiophene-5′′-yl)-pyrrolo[3,4-c]pyrrolo-1,4-dione-6-yl]-thiophene-5-yl}-2,1,3-benzothiadiazole (bis-DPP) blended with [70]PCBM^17^.

### Recombination and extraction rates

First, we present an approximate treatment of recombination and extraction of charge carriers from an organic solar cell. Next, numerical simulations are used to show that this treatment, although approximate, can explain the different FFs.

For simplicity, we consider a solar cell with uniform absorption across the active layer and constant electron and hole mobility; it has been shown that including an optical profile in the modelling of the device does not significantly change the results for active layer thinner than 300 nm^39^. As one cannot give a single value for the voltage at the MPP, we consider short-circuit conditions instead. On average, electrons have to travel half the active layer thickness (*L*), to reach the cathode. Thus, their extraction rate *k*_ex_ can be approximated as


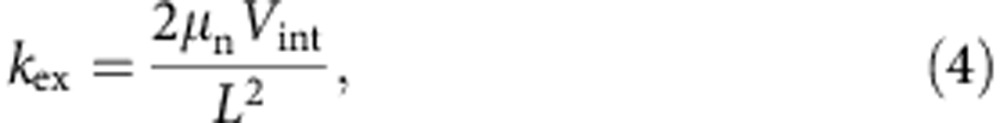


where *V*_int_ is the internal voltage. The internal voltage is related to the LUMO level of the acceptor and the HOMO level of the donor by


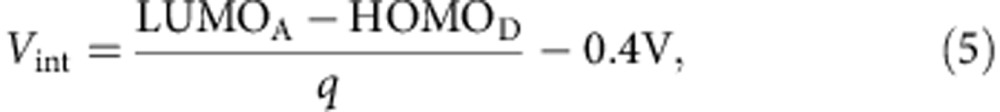


where we subtract 0.4 V to account for band bending^40^. In case these levels are not known, *V*_int_ is approximated by the *V*_OC_ at 1 sun intensity.

As can be seen in Fig. 6b of ref. 22, most of the bimolecular recombination occurs near the electrodes; there, the density of one of the two charge carriers is large due to the presence of the ohmic contact and this results in a high probability for charges of opposite sign to meet each other and recombine. This is also the case in the simulations in this study, which assumes the contacts to be ohmic. Near the cathode, for example, the electron density due to the presence of the contact overwhelms the density of photogenerated electrons. The density of holes, on the other hand, is much lower and stems from the rate at which they are generated and extracted. Because of this, the density of holes is very small at the cathode and increases with increased distance from the cathode. If holes are generated at a volume rate *G* and flow towards the anode at a speed *μ*_p_*V*/*L*, then the hole density close to the cathode is approximately


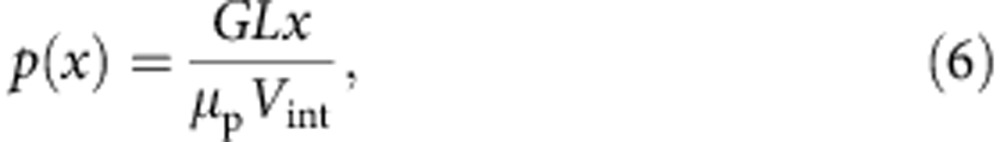


where *x* is the distance from the cathode. If recombination is very strong, equation (6) will overestimate the density of holes. The average density of holes at the cathode side of the layer (0≤*x*≤*L*/2) equals


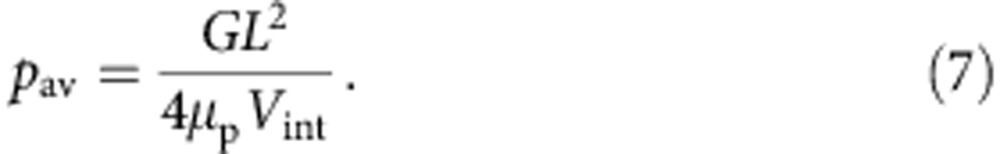


To verify whether this equation holds, we have performed numerical simulations (see Supplementary Fig. 2).

The number of recombination events per volume and time is given by


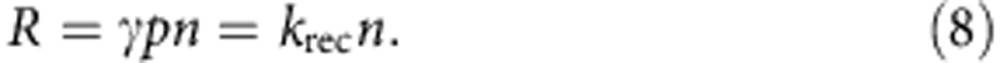


Thus, the recombination rate *k*_rec_ of electrons with holes near the cathode equals


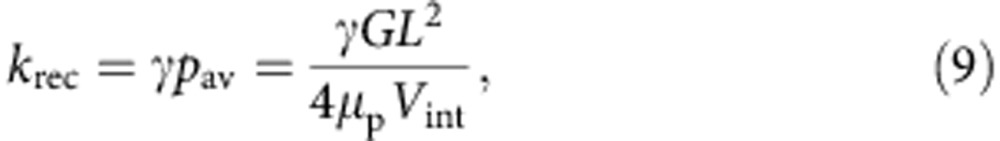


where *p*_av_ is given by equation (7). Owing to the symmetry of electrons and holes, a similar reasoning applies to recombination at the anode. The ratio of extraction-to-recombination, *θ*, is defined as


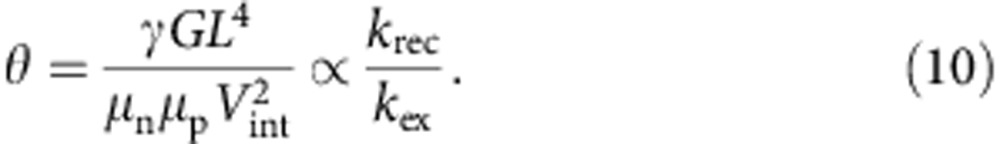


Thus, two solar cells with similar *θ* are expected to have similarly large recombination losses. Our hypothesis is that the competition between recombination and extraction of charges, as quantified by *θ*, governs the FF of organic solar cells.

### Results of the numerical simulations

To obtain a full picture of the factors determining the FF, we simulate *J–V* characteristics with a large range of FFs. The values of the parameters used for the simulations are varied, within the interval specified in Table 1, to obtain a uniform distribution of log(*θ*) over the range *θ*=10^−6^–10^3^. We get a set of resulting *J–V* characteristics with FFs that spans from 0.25–0.87.

**Figure 3:**
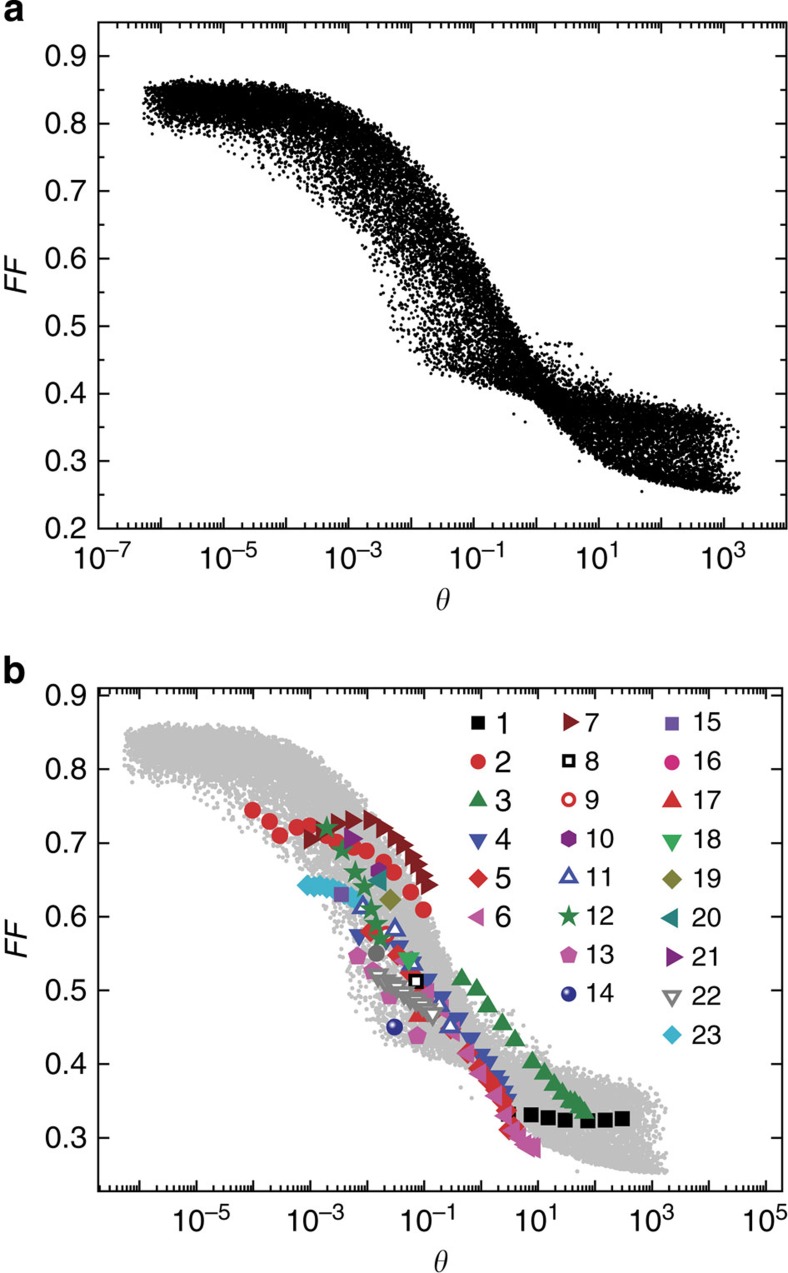
Dependency of the FF on the parameter *θ*. (**a**) Simulated FF*–θ* points; (**b**) FF versus *θ* for the simulated (small, grey symbols) and the experimental data (big, colour symbols). The empty symbols represent systems for which the generation of charges has been shown to be field dependent. The meaning of the numbers in the legend is explained in Table 2.

The FFs of the simulated *J–V* curves are plotted against *θ* in Fig. 3a. Remarkably, the simulated FFs follow a clear pattern when plotted against *θ*; for lower recombination/extraction ratios, the highest values of FF are achieved; an exponential decrease of the FF is observed when the recombination losses become more important; and a plateau at low FF values is reached when the recombination rate is significantly higher than the extraction rate. This finding makes it possible to predict the effect of simultaneously changing multiple parameters on the FF.

### Experimental results

The parameter *θ* depends on the properties of the materials and of the devices. Experimentally, it is possible to achieve solar cells with *θ* spanning a wide range by producing devices based on different blends, by varying the processing conditions, or by measuring the same device in different conditions of temperature and incident light. In particular, *θ* can be made to change over orders of magnitude by changing the intensity of the incident light. Furthermore, the thickness of the active layer can be varied to investigate the behaviour of the same blend over a range of *θ.*

To determine *θ*, it is necessary to know the mobilities of holes and electrons. The charge transport in PDPP5T:[70]PCBM and PTB7:[70]PCBM blends is characterized by measuring the *J–V* characteristics of single carrier devices (see Supplementary Fig. 3). The current in such devices is limited by space charge and is directly proportional to the mobility. The mobility is determined by fitting (see Supplementary Fig. 3 for details). In addition to the mobilities, the strength of the bimolecular recombination *γ* has to be measured. For PDPP5T:[70]PCBM and PTB7:[70]PCBM blends, we use a method proposed by Wetzelaer *et al*.^41^, which consists of determining *γ*_pre_ from the steady-state characterization of single- and double-carrier devices, by the relation


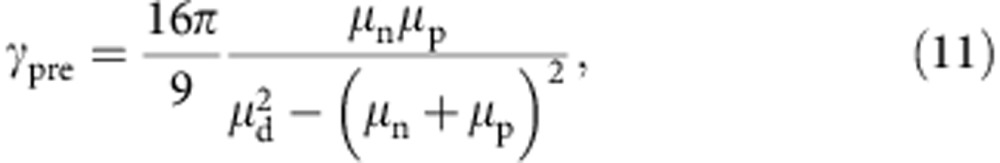


where *μ*_d_ is the effective double-carrier mobility obtained by fitting the dark current of the solar cells (see Supplementary Fig. 4). Next, we calculate *γ* using equations (2) and (3).

Recombination rates and carrier mobilities in P3HT:[70]PCBM and in polymer:polymer systems are measured by the use of time-delayed collection field (TDCF) measurements^21^. In TDCF, charge carriers are photogenerated with a short laser pulse under constant pre-bias conditions and subsequently extracted with a constant high reverse voltage, which is applied after a defined delay time. The bimolecular recombination coefficients *γ* are obtained by applying an iterative calculation of the charges that are collected after varying delay times with respect to the charge that has been extracted during the pre-bias^11^,^21^. Charge-carrier mobilities can be determined from extrapolating the photocurrent decay or by fitting the whole current transients that are recorded in TDCF measurements by means of numerical drift-diffusion simulations^11^,^21^,^30^ (see Supplementary Figs 5 and 6).

The values of *γ* for MDMO-PPV:PCBM^16^, PBnDT-(X)TAZ^23^, mono-DPP:[70]PCBM and bis-DPP:[70]PCBM^17^ are taken from the literature. Table 2 contains *μ*_n_, *μ*_p_ and *γ* for all the systems analysed.

For the PDPP5T:[70]PCBM and P3HT:[70]PCBM systems, *θ* is varied by changing the processing conditions and by measuring at different light intensities. For PDPP5T:[70]PCBM blends, we obtain different values of electron mobility by changing the concentration of fullerene derivative in the blend; the mobility of holes is rather insensitive to the concentration of [70]PCBM in the range we investigated; thus, by changing the composition of these blends we move from a situation of balanced transport to a large difference between the mobilities of the two carriers. For P3HT:[70]PCBM blends as well, we analyse devices with both balanced and unbalanced charge transport, as the mobility of holes in P3HT:[70]PCBM blends can be greatly enhanced by thermal annealing, leaving the electron mobility rather unchanged^42^. Moreover, a strong reduction of the strength of the bimolecular recombination in P3HT:[70]PCBM blends is observed on annealing.

For PTB7:[70]PCBM, different solvents have been used. When chlorobenzene is used as a solvent, the deposition of the blend results in a layer with large-scale phase separation; employing *ortho*-dichlorobenzene or adding diiodooctane as a co-solvent reduces the length scale of space separation^43^. In addition to the difference in the recombination strength when different solvents are used, the different morphology of the active layer influences the generation rate of charges. In the device processed by adding DIO as a co-solvent, the generation of charges has been shown to be field independent, in contrast to the devices processed without co-solvent^44^.

The data series taken from the literature for MDMO-PPV:PCBM^16^ and for PBnDt-(X)TAZ:PCBM^23^ were collected from different samples, all measured under illumination at 1 sun intensity. The MDMO-PPV:PCBM series compares devices with different active layer thickness^16^; the PBnDt-(X)TAZ:PCBM data points regard devices in which the polymer has a different degree of fluorination. As shown in ref. 23, the performance of these blends is limited by the extraction of charges; increasing the fluorination enhances the mobility of holes, leaving generation and recombination rates unchanged.

For the P3HT:P(NDI2OD-T2), we have two different samples in which the deposition of the active layer occurred under different conditions; for each, we present the results of measurements performed when varying the illumination intensity. When the drying speed is increased by heating the sample immediately after spin casting the blend, the bimolecular recombination is reduced by one order of magnitude^30^. As the mobilities of electrons and holes do not change significantly, this increase in FF on faster drying may be due to the reduction of *γ*. The other data from polymer:polymer systems compare the results obtained with two different acceptor polymers.

The literature data for the small-molecule systems mono-DPP:[70]PCBM and bis-DPP:[70]PCBM^17^ were taken from two devices whose FFs have been measured at different illumination intensities. The difference in FF between the two systems is mostly given by the lower value of hole mobility that characterizes the mono-DPP system^17^.

For every experimental value of the FF obtained from the measurement of the *J–V* curves of the solar cells, *θ* is calculated according to equation (9). For P3HT:[70]PCBM blends, the generation rate of free charges (*G*) is measured by means of TDCF. For all the other systems, *G* is calculated from the short circuit current,


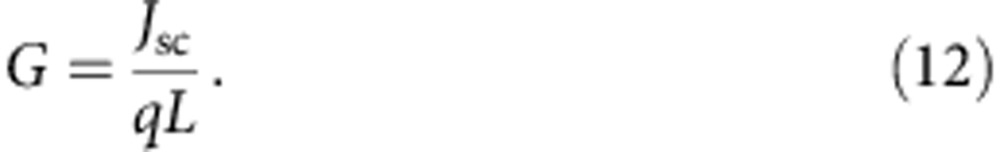


For the internal voltage, we use equation (5) for the donor:acceptor systems of which the HOMO and LUMO levels are known. For the other systems, we approximate *V*_int_ by using the value of *V*_oc_ measured at 1 sun.

We then plot the experimental FF*–θ* curves for every device and compare it with the simulated data in Fig. 3b. All the experimental data follow the same trend of the simulated points; in general, lower *θ* results in higher FF. The rapid decrease of the FF for values of *θ* in the range between 10^−3^ and 1 is confirmed by the experiments. This agreement between experiments and simulations confirms that the dependency of the FF on the ratio of the rates of recombination and extraction of free charges can be described by using the parameter *θ*. In PTB7:[70]PCBM devices processed without co-solvent, in MDMO-PPV:PCBM and in mono-DPP:[70]PCBM devices (empty dots in Fig. 3b), the generation of charges is field dependent^16^,^17^,^44^. However, this field dependency of the generation appears to have little influence on our results; thus, for these systems as well, the FF is determined by the competition between extraction and non-geminate recombination as quantified by *θ*.

## Discussion

**Figure 4:**
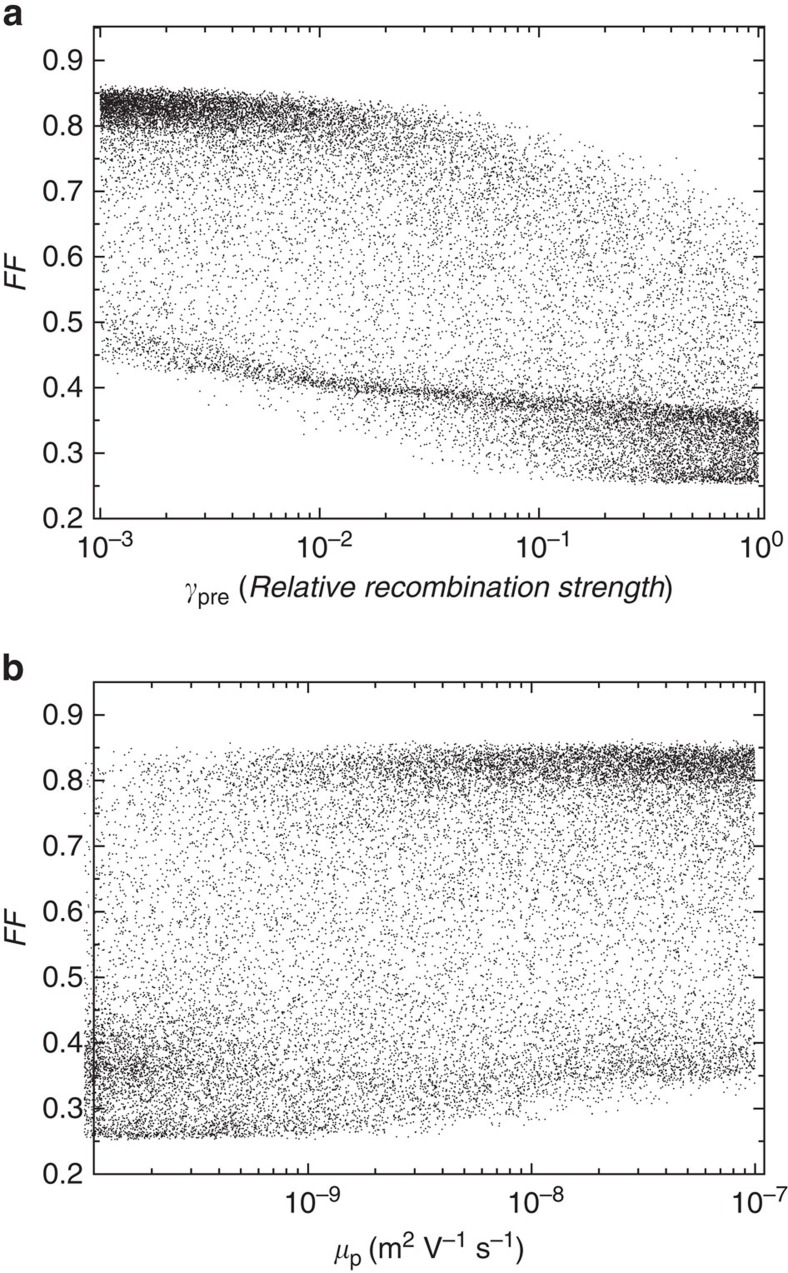
Dependency of the FF on the recombination prefactor and on the hole mobility. FF versus *γ*_pre_ (**a**) and *μ*_p_ (**b**) for the simulated data.

The overall dependency of FF on *θ* is clearly visible in Fig. 3a,b, which demonstrates, both theoretically and experimentally, that the ratio of the recombination and extraction rates of free charges determines the FF. This finding can be used to predict the effect of changing multiple parameters simultaneously. By contrast, when the FFs of the simulated *J–V* curves are plotted against any of the individual parameters listed in Table 1, scattered clouds of points are obtained. For example, in Fig. 4a,b FF is plotted against the reduction prefactor *γ*_pre_ for the bimolecular recombination and against the mobility of holes. The dependency of FF on the other parameters is shown in Supplementary Fig. 7. Clearly, these individual parameters can not be used to predict the FF of organic solar cells.

Having expressed the ratio of the recombination and extraction rates as in equation (10), we can explain why FFs change significantly with light intensity, thickness and material properties. On varying the light intensity, the extraction rate of charges is not modified, while the recombination rate changes according to equation (9). At low intensities, the density of charge in the active layer is low and the recombination is slow. Therefore, a decrease of *θ* is achieved when the light intensity is reduced, keeping the other parameters constant. As shown in Fig. 3a, moving towards lower *θ* is beneficial for the FF. This general pattern is valid for all the data measured near 1 sun intensity, over a range of *θ* that spans six orders of magnitude (from 10^−3^ to 10^3^). The 1:2 wt ratio blend of PDPP5T:[70]PCBM (combination 7 in Fig. 3b) does not represent an exception; the experimental data for this blend measured close to 1 sun follows the simulated trend. However, this blend shows a deviation from the expected behaviour at low light intensity. We speculate that this deviation is due to the presence of another recombination pathway, such as trap-assisted recombination, which becomes important at low light intensities.

Increasing the thickness enhances the absorption of light, consequently increasing the charge density and the recombination rate. At the same time, if the active layer gets thicker the extraction of charges becomes slower, as they need more time to leave the active layer. For this reason, *θ* increases with increasing *L*, resulting in lower FFs.

The internal voltage has an influence on both extraction and recombination rates. The extraction of charges is faster if these move in a stronger electric field; thus, increasing *V*_int_ reduces the extraction rate and at the same time a larger *V*_int_ lowers the carrier density at the contacts (equation (7)), making the recombination rate smaller.

The reduction prefactor *γ*_pre_ for the recombination strength acts only on the recombination rate, leaving the extraction rate unchanged. In general, a reduction of *θ*, and hence an improvement of FF, is observed when *γ*_pre_ is reduced, keeping the other parameters constant.

Finally, the mobilities of electrons and holes affect both *k*_rec_ and *k*_ex_. Increasing the mobilities enhances the strength of the bimolecular recombination *γ*, expressed in equation (3). However, faster charge carriers imply a faster extraction of charges and a lower charge density near the contact. Thus, the overall effect of increasing the mobilities of holes and electrons is a reduction of *θ*.

Our result and the comparison of all the experimental data presented in this study with the simulated data support the generality of our conclusion: whether *θ* is varied by changing the thickness of the active layer, the combination of donor and acceptor materials, the solvent or the measurement conditions, the ratio of extraction and recombination times appears to be strongly related to the FF. The generality of our finding is further supported by the fact that experimental data have been collected using both steady-state and transient techniques, and from very different organic solar cells. For polymer:fullerene, polymer:polymer and small-molecule devices, the underlying factors that govern FF appear to be the same. We note that Fig. 3 represents the upper limit for the FF; a reduction of FF may be observed if the photogeneration of charges is strongly dependent on the electric field^10^,^45^,^46^ or, in case of poor electrical contacts, on the electrodes^47^. However, both these limiting factors are in general not present in most efficient donor:acceptor systems. We also note that implementing a near-zero surface recombination velocity in our model does not significantly modify the resulting FF versus *θ* (see Supplementary Fig. 1).

It is not uncommon that when trying to improve the efficiency of organic solar cells by tuning the processing or replacing one of the constituent materials, the transport and recombination change in non-trivial ways. For example, when comparing combination 13 with combination 15, the recombination rate decreases while the charge transport deteriorates. A priori, it is not clear whether this would result in a decrease or increase of the FF. The resulting values of *θ* (7.6 × 10^−2^ and 3.5 × 10^−3^ for combination 13 and 15, respectively) indicate that the reduction of the recombination rate outweighs the deterioration of charge transport in this case. Based on Fig. 3b, relatively small changes in *θ* are expected to significantly change FF. Indeed, the FF improved from 0.43 to 0.63.

Recently, Proctor *et al*.^18^ have proposed that to get FFs of 0.65 or higher, the mobilities should be at least 10^−7^ m^2^ V^−1^ s^−1^. From our findings, a general guideline for the targeted improvement of the FF of organic solar cells can be defined. Aiming to obtain FF of 0.8 or higher, a device with *θ* smaller than 10^−4^ has to be achieved (see Fig. 3a). Fig. 4b suggests that such high FFs could be achieved at relatively low mobilities. However, this graph contains data for the whole range of parameters as listed in Table 1, including low light intensities and thin active layers. For a typical device at 1 sun illumination, we assume *L*=100 nm, *G*=10^28^ m^−3^ s^−1^ and *V*_int_=1 V, which leaves the ratio *γ*/(*μ*_p_*μ*_n_) as the only determinant for *θ*, and hence for the FF. From equation (9), it follows that for such a device it would be necessary to improve the transport and recombination properties of the blend so that *γ*/(*μ*_p_*μ*_n_) <10^−4^ V^2^ s m^−1^. It should be noted that extreme differences between electron and hole mobility should be avoided even if *θ*<10^−4^, as this leads to the build-up of space charge^48^. For example, if the mobilities are 10^−7^ m^2^ V^−1^ s^−1^, *γ* should be smaller than 10^−18^ m^3^ s^−1^, which is equivalent to *γ*_pre_ <8 × 10^−4^, to get an FF of at least 0.8. This highlights the importance of controlling recombination besides improving charge transport.

In conclusion, we have shown that the competition between charge recombination and extraction, which governs the FF in the whole range of 0.26–0.74, can be quantified by the parameter *θ*, which is the ratio of the rates of recombination and extraction of free charges. We have shown a relationship between FF and *θ*, which is valid for a large number of donor:acceptor combinations, under the assumption of ohmic contacts. Our conclusion is supported by experimental data collected from polymer:fullerene, polymer:polymer and small-molecule devices, which we characterized with steady-state and transient extraction techniques. The field dependence of the charge generation in some of the systems that we considered does not have a significant effect on the results. Further evidence for the clear relationship between FF and *θ* comes from drift-diffusion simulations of organic solar cells performing varying charge-carrier mobilities, recombination rate, light intensity, energy levels and active-layer thickness over a wide range. The results presented here provide new insights into the physical phenomena governing the FF of organic solar cells and help explain why the FFs change significantly with material properties, light intensity and thickness. The relationship between FF and *θ* shown by this work offers an approach for targeted improvements of FF. In particular, this relationship can be used to rationalize the effect on FF of simultaneously changing multiple parameters. In addition, we indicated in which way recombination and transport properties of a blend should be modified for a device with given thickness, generation rate and internal voltage, to optimize its FF.

## Methods

### Device fabrication

*PDPP5T:[70]PCBM*. Solar cells were fabricated on glass substrates prepatterned with indium tin oxide (ITO). The substrates were thoroughly cleaned by washing with detergent solution and ultrasonication in acetone and isopropyl alcohol, and subsequent ultraviolet–ozone treatment. A 60-nm-thick film of poly(3,4-ethylenedioxythiophene):poly(styrenesulfonic acid) (PEDOT:PSS; VP AI4083, H.C. Stark) was then spin cast on the substrate. PDPP5T:[70]PCBM blends were deposited by spin casting from chloroform/*ortho*-dichlorobenzene (5 vol%) solutions in N_2_ atmosphere. For PTB7:[70]PCBM, different solvents were used (see Table 2). After drying of the polymer:fullerene films at room temperature (RT), a cathode of LiF(1 nm)/Al(100 nm) was applied via thermal evaporation, defining an active area of 4 mm^2^.

Single-carrier devices were fabricated on glass substrates cleaned following the same procedure. The polymer:fullerene films were sandwiched between Cr(1 nm)/Au(30 nm)/PEDOT:PSS and Pd(20 nm)/Au(80 nm) to create hole-only devices; for the fabrication of electron-only devices, Al(30 nm) and LiF(1 nm)/Al(100 nm) were selected as bottom and top contact, respectively. All the metal layers have been deposited via thermal evaporation.

*P3HT:[70]PCBM*. Solar cells were fabricated on structured ITO glass substrates (Optrex) coated with a 60-nm layer of PEDOT:PSS (Clevios AI 4083). P3HT Sepiolid P200 (purchased from BASF) and [70]PCBM (purchased from Solenne) were separately dissolved in chloroform, then mixed to a 1:1 (by weight) solution with a concentration of 25 g l^−1^and subsequently spin coated at 1,000 r.p.m., yielding an active-layer thickness of 200 nm. Devices referred to as ‘annealed' were heated at 150 °C for 15 min, directly after spin coating. Finally, 20 nm Sm and 100 nm Al were thermally evaporated through shadow masks, defining an active area of 1 mm^2^.

*P3HT:P(NDI2OD-T2) and P3HT:P(PDI2OD-T2)*. Solar cells were prepared on glasses with structured ITO electrodes. The samples were washed in Acetone, detergent solution, deionized water and isopropanol, and were plasma treated before spin coating of 60 nm PEDOT:PSS. The polymer blends were spin cast from a 1:1 xylene:chloronaphthalene solution with a donor/acceptor ratio of 1:0.75 and a concentration of 40 g l^−1^ for P3HT:P(NDI2OD-T2) and a ratio of 2:1 and a concentration of 33 g l^−1^ for P3HT:P(PDI2OD-T2). The P3HT:P(NDI2OD-T2) and P3HT:P(PDI2OD-T2) films were spun for 5 s and subsequently dried for 2 min at 200 °C and 180 °C, respectively. The P3HT:P(NDI2OD-T2) films that were slowly dried have instead been kept under vacuum at RT for 16 h after spin coating. The solar cells have been finalized by evaporating 20 nm Sm and 100 nm Al.

*POPT:P(NDI2OD-T2) and POPT:P(PDI2OD-T2)*. The POPT:P(NDI2OD-T2) and POPT:P(PDI2OD-T2) solar cells were prepared from a 4:1 and 9:1 dichlorobenzene:chloronaphthalene solution with concentrations of 15 and 24 g l^−1^, respectively. The donor/acceptor ratio of POPT:P(NDI2OD-T2) and POPT:P(PDI2OD-T2) was 2:1 and 1.5:1, respectively. POPT:P(NDI2OD-T2) films were annealed at 80 °C for 2 min. POPT:P(PDI2OD-T2) films were dried at RT and subsequently put on a hot plate at 150 °C for 10 min.

### Device characterization

*Steady-state measurement of charge mobilities and recombination*. Electrical measurements of the *J–V* characteristics of PDPP5T:[70]PCBM and PTB7:[70]PCBM devices were performed using a computer-controlled Keithley source meter in N_2_ atmosphere. The measurements under illumination were done using a Steuernagel SolarConstant 1200 metal halide lamp; a silicon reference cell was employed to correct for spectral mismatch with AM1.5G spectrum and set the intensity of the lamp to 1 sun. Light-intensity dependency was measured by varying the intensity of the light with a series of neutral density filters.

*Time-delayed collection field*. In the TDCF experiments, pulsed excitation (5.5 ns pulse width, 500 Hz repetion rate) was realized with a diode-pumped, Q-switched neodymium-doped yttrium aluminium garnet laser (NT242, EKSPLA). The photogenerated charge carriers were extracted by applying a high rectangular voltage pulse with a pulse generator (Agilent 81150A) in reverse direction. The current through the device was measured with a Yokogawa DL9140 oscilloscope via a 50-Ω input resistor.

## Author contributions

L.J.A.K. conceived the project. D.B. and I.d.C.P. performed the experiments on PDPP5T:[70]PCBM and PTB7:[70]PCBM systems, and analysed the data. J.K. and S.R. performed the experiments on P3HT:[70]PCBM and the polymer:polymer blends, and analysed the data. D.N. supervised their research. M.T. synthesized PDPP5T. L.J.A.K. performed the simulations. D.B. and L.J.A.K. wrote the manuscript with contributions from all authors.

## Additional information

**How to cite this article**: Bartesaghi, D. *et al*. Competition between recombination and extraction of free charges determines the fill factor of organic solar cells. *Nat. Commun.* 6:7083 doi: 10.1038/ncomms8083 (2015).

## Supplementary Material

Supplementary Information Supplementary Figures 1-7, Supplementary Note 1 and Supplementary References

## Figures and Tables

**Figure 1:**
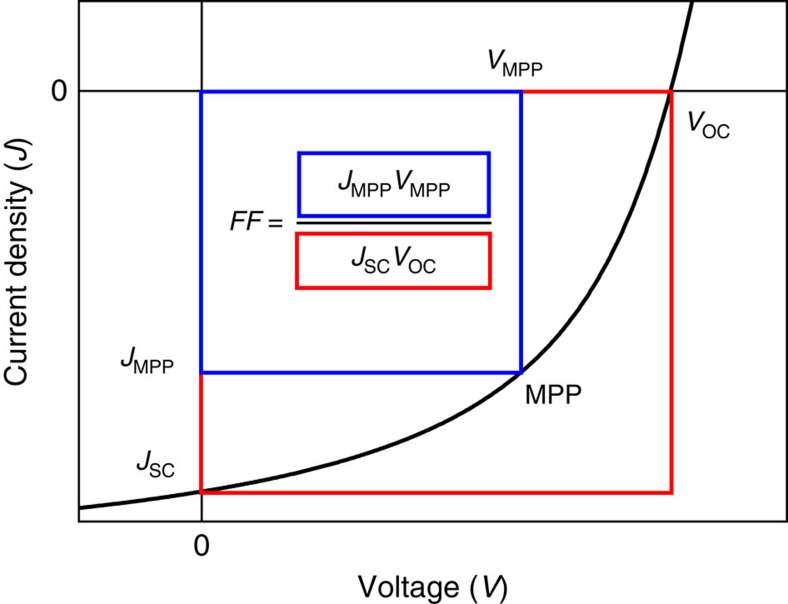
*J–V* characteristic of a solar cell. The MPP is the voltage at which the product *|JV*| is at its maximum.

**Table 1:** Parameters used in the drift-diffusion simulations.

**Symbol**	**Description**	**Range**
*L*	Thickness	60–260 nm
LUMO_A_–HOMO_D_	Effective bandgap	1.0–1.3 eV
*μ*_p_	Hole mobility	1 × 10^−10^−1 × 10^−7^ m^2^ V^−1^ s^−1^
*μ*_n_	Electron mobility	1 × 10^−10^−1 × 10^−7^ m^2^ V^−1^ s^−1^
*γ*_pre_	Recombination pre-factor	1 × 10^−3^−1
*G*	Generation rate of free charges	1 × 10^25^−1 × 10^28^ m^−3^ s^−1^

HOMO_D_, highest occupied molecular orbital of the donor; LUMO_A_, lowest unoccupied molecular orbital of the acceptor.

**Table 2:** Materials parameters.

**Number**	**Material**	***μ***_**n**_**(m**^**2**^ **V**^−**1**^ **s**^−**1**^)	***μ***_**p**_**(m**^**2**^ **V**^−**1**^ **s**^−**1**^)	***γ*****(m**^**3**^ **s**^−**1**^)	***V***_**int**_(***V***)
1	P3HT:[70]PCBM as cast	1.7 × 10^−8^	1.0 × 10^−10^	1.1 × 10^−17^	0.58
2	P3HT:[70]PCBM annealed	1.8 × 10^−7^	4.0 × 10^−8^	1.1 × 10^−18^	0.58
3	PDPP5T:[70]PCBM 2:1^a^	2.4 × 10^−11^	1.2 × 10^−7^	4.9 × 10^−17^	0.583^b^
4	PDPP5T:[70]PCBM 1:1 (295 K)	1.1 × 10^−8^	3.0 × 10^−7^	7.3 × 10^−16^	0.566^b^
5	PDPP5T:[70]PCBM 1:1 (255 K)	1.4 × 10^−9^	9.5 × 10^−8^	5.7 × 10^−17^	0.624^b^
6	PDPP5T:[70]PCBM 1:1 (215 K)	9.2 × 10^−11^	1.9 × 10^−8^	3.6 × 10^−18^	0.675^b^
7	PDPP5T:[70]PCBM 1:2	3.1 × 10^−7^	2.9 × 10^−7^	5.4 × 10^−16^	0.556^b^
8	PTB7:[70]PCBM in CB	8.0 × 10^−8^	5.4 × 10^−8^	1.5 × 10^−16^	0.757^b^
9	PTB7:[70]PCBM in oDCB	3.5 × 10^−8^	3.0 × 10^−8^	8.3 × 10^−18^	0.74^b^
10	PTB7:[70]PCBM in CB/DIO	2.3 × 10^−8^	1.3 × 10^−8^	1.7 × 10^−18^	0.735^b^
11	MDMO-PPV:PCBM^16^	2.0 × 10^−7^	3.0 × 10^−8^	6.0 × 10^−17^	0.8
12	P3HT:P(NDI2OD-T2) (fast dried)	4.6 × 10^−7^ c	1.0 × 10^−7^ c	7.3 × 10^−18^	0.5
13	P3HT:P(NDI2OD-T2) (slow dried)	3.8 × 10^−7^ c	2.5 × 10^−7^ c	6.6 × 10^−17^	0.5
14	P3HT:P(PDI2OD-T2)	3.0 × 10^−7^ c	1.1 × 10^−7^ c	8.0 × 10^−18^	0.48
15	POPT:P(NDI2OD-T2)	2.0 × 10^−7^ c	3.5 × 10^−8^ c	9.1 × 10^−19^	0.48
16	POPT:P(PDI2OD-T2)	1.4 × 10^−7^ c	3.7 × 10^−8^ c	5.5 × 10^−18^	0.44
17	PBnDt-(X)TAZ:PCBM (F00)^23^ ^d^	4.0 × 10^−7^	1.71 × 10^−8^	1.0 × 10^−17^	0.731^b^
18	PBnDt-(X)TAZ:PCBM (F25)^23^	4.0 × 10^−7^	2.76 × 10^−8^	1.0 × 10^−17^	0.742^b^
19	PBnDt-(X)TAZ:PCBM (F50)^23^	4.0 × 10^−7^	5.64 × 10^−8^	1.0 × 10^−17^	0.764^b^
20	PBnDt-(X)TAZ:PCBM (F75)^23^	4.0 × 10^−7^	8.02 × 10^−8^	1.0 × 10^−17^	0.78^b^
21	PBnDt-(X)TAZ:PCBM (F100)^23^	4.0 × 10^−7^	1.22 × 10^−8^	1.0 × 10^−17^	0.797^b^
22	mono-DPP:[70[PCBM^17^	1.0 × 10^−7^	2.0 × 10^−9^	5.3 × 10^−17^	0.75
23	bis-DPP:[70]PCBM^17^	1.5 × 10^−7^	3.4 × 10^−8^	2.6 × 10^−17^	0.5

bis-DPP, 4,7-bis{2-[2,5-bis(2-ethylhexyl)-3-(5-hexyl-2,2′:5′,2′′-terthiophene-5′′-yl)-pyrrolo[3,4-c]pyrrolo-1,4-dione-6-yl]-thiophene-5-yl}-2,1,3-benzothiadiazole; CB, chlorobenzene; DIO, diiodooctane; FTAZ, fluorinated analogue of HTAZ; HTAZ, 2-alkyl-benzo[d][1,2,3]triazole; MDMO-PPV, poly(2-methoxy-5-(3′,7′-dimethyl octyloxy)-*p*-phenylene vinylene); mono-DPP, 2,5-di-(2-ethylhexyl)-3,6-bis-(5′′-n-hexyl-[2,2′,5′,2′′]terthiophen-5-yl)-pyrrolo[3,4-c]pyrrole-1,4-dione; *oDCB, ortho*-dichlorobenzene; PDPP5T, diketopyrrolopyrrole-quinquethiophene alternating copolymer; PH3T, poly(3-hexylthiophene); P(NDI2OD-T2), poly([N,N′-bis(2-octyldodecyl)-naphtalene-1,4,5,8-bis(dicarboximide)-2,6-diyl]-alt-5,5′-(2,2′-bithiophene)); POPT, poly[3-(4-octylphenyl)thiophene]; P(PDI2OD-T2), perylene-based analogue of P(NDI2OD-T2); PTB7, polythieno[3,4-b]-thiophene-co-benzodithiophene; [70]PCBM, [6,6]-phenyl-C_71_-butyric acid methyl esther.

^a^The ratio PDPP5T:[70]PCBM is expressed in w/w.

^b^The internal voltage for these systems is approximated by using the value for *V*_oc_ measured at 1 sun.

^c^For this system, it is not known which mobility corresponds to which charge carrier. However, all our equations are symmetric in electrons and holes; thus, we assume that the electrons have the higher mobility. This assumption does not affect our results.

^d^Nomenclature for PBnDt-(X)TAZ:PCBM: the number after F indicates the molar ratio of FTAZ in (X)TAZ, for example, F25 is the polymer made with a feed ratio of HTAZ:FTAZ 3:1.

## References

[b1] Li G. . High-efficiency solution processable polymer photovoltaic cells by self-organization of polymer blends. Nat. Mater. 4, 864–868 (2005).

[b2] Liang Y. . For the bright future – bulk heterojunction polymer solar cells with power conversion efficiency of 7.4%. Adv. Mater. 22, E135–E138 (2010). 20641094 10.1002/adma.200903528

[b3] He Z. . Enhanced power-coversion efficiency in polymer solar cells using an inverted device structure. Nat. Photonics 6, 591–595 (2012).

[b4] Liu Y. . Aggregation and morphology control enables multiple cases of high-efficiency polymer solar cells. Nat. Commun. 5, 5293 (2014). 25382026 10.1038/ncomms6293 PMC4242436

[b5] Monestier F. . Modeling the short-circuit current density of polymer solar cells based on P3HT:PCBM blend. Sol. Energy Mater. Sol. Cells 91, 405–410 (2007).

[b6] Burkhard G. F., Hoke E. T. & McGehee M. D. Accounting for interference, scattering, and electrode absorption to make accurate internal quantum efficiency measurements in organic and other thin solar cells. Adv. Mater. 22, 3293–3297 (2010). 20517871 10.1002/adma.201000883

[b7] Gadisa A., Svensson M., Andersson M. R. & Inganäs O. Correlation between oxidation potential and open-circuit voltage of composite solar cells based on blends of polythiophenes/fullerene derivative. Appl. Phys. Lett. 84, 1609–1611 (2004).

[b8] Vandewal K., Tvingstedt K., Gadisa A., Inganäs O. & Manca J. V. On the origin of the open-circuit voltage of polymer-fullerene solar cells. Nat. Mater. 8, 904–909 (2009). 19820700 10.1038/nmat2548

[b9] Koster L. J. A., Mihailetchi V. D., Ramaker R. & Blom P. W. M. Light intensity dependence of open-circuit voltage of polymer:fullerene solar cells. Appl. Phys. Lett. 86, 123509 (2005).

[b10] Dibb G. F. A., Jamieson F. C., Maurano A., Nelson J. & Durrant J. R. Limits om the fill factor in organic photovoltaics: distinguishing nongeminate and geminate recombination mechanisms. J. Phys. Chem. Lett. 4, 803–808 (2013). 10.1021/jz400140p 26281936

[b11] Albrecht S. . Fluorinated copolymer PCPDTBT with enhanced open-circuit and reduced recombination for highly efficient polymer solar cells. J. Am. Chem. Soc. 134, 14932–14944 (2012). 22861119 10.1021/ja305039j

[b12] Lakhwani G., Rao A. & Friend R. H. Bimolecular recombination in organic photovoltaics. Annu. Rev. Phys. Chem. 65, 557–581 (2014). 24423376 10.1146/annurev-physchem-040513-103615

[b13] Mauer R., Howard I. A. & Laquai F. Effect of nongeminate recombination on fill factor in polythiophene/methanofullerene organic solar cells. J. Phys. Chem. Lett. 1, 3500–3505 (2010).

[b14] Kirchartz T., Agostinelli T., Campoy-Quilles M., Gong W. & Nelson J. Understanding the thickness-dependent performance of organic bulk heterojunction solar cells: the influence of mobility, lifetime, and space charge. J. Phys. Chem. Lett. 3, 3470–3475 (2012). 10.1021/jz301639y 26290974

[b15] Baumann A., Rauh J., Deibel C. & Dyakonov V. A new approach for probing the mobility and lifetime of photogenerated charge carriers in organic solar cells under real operating conditions. Adv. Mater. 24, 4381–4386 (2012). 22760962 10.1002/adma.201200874

[b16] Lenes M., Koster L. J. A., Mihailetchi V. D. & Blom P. W. M. Thickness dependence of the efficiency of polymer:fullerene bulk heterojunction solar cells. Appl. Phys. Lett. 88, 243502 (2006).

[b17] Proctor C. M., Kim C., Neher D. & Nguyen T. -Q. Nongeminate recombination and charge transport limitations in diketopyrrolopyrrole-based solution-processed small molecule solar cells. Adv. Funct. Mater. 23, 3584–3594 (2013).

[b18] Proctor C. M., Love J. A. & Nguyen T. -Q. Mobility guidelines for high fill factor solution-processed small molecule solar cells. Adv. Mater. 26, 5957–5961 (2014). 25047697 10.1002/adma.201401725

[b19] Zhang Y., Dang X. -D., Kim C. & Nguyen T. -Q. Effect of charge recombination on the fill factor of small molecule bulk heterojunction solar cells. Adv. Energy Mater. 1, 610–617 (2011).

[b20] Wu L., Zang H., Hsiao Y. -C., Zhang X. & Hu B. Origin of the fill factor loss in bulk-heterojunction organic solar cells. Appl. Phys. Lett. 104, 153903 (2014).

[b21] Kniepert J., Lange I., van der Kaap N. J., Koster L. J. A. & Neher D. A conclusive view on charge generation, recombination, and extraction in as-prepared and annealed P3HT:PCBM blends: combined experimental and simulation work. Adv. Energy Mater. 4, 1301401 (2014).

[b22] Koster L. J. A., Smits E. C. P., Mihailetchi V. D. & Blom P. W. M. Device model for the operation of polymer/fullerene bulk heterojunction solar cells. Phys. Rev. B 72, 085205 (2005).

[b23] Li W. . Mobility-controlled performance of thick solar cells based on fluorinated copolymers. J. Am. Chem. Soc. 136, 15566–15576 (2014). 25341026 10.1021/ja5067724

[b24] MacKenzie R. C. I., Shuttle C. G., Chabinyc M. L. & Nelson J. Extracting microscopic device parameters from transient photocurrent measurements. Adv. Energy Mater. 2, 662–669 (2012).

[b25] Tress W., Leo K. & Riede M. Influence of hole-transport layers and donor materials on open-circuit voltage and shape of *I-V* curves of organic solar cells. Adv. Funct. Mater. 21, 2140–2149 (2011).

[b26] Hwang I., McNeill C. R. & Greenham C. Drift-diffusion modelling of photocurrent transients in bulk heterojunction solar cells. J. Appl. Phys. 106, 094506 (2009).

[b27] Langevin P. Recombinaison et mobilites des ions dans les gaz. Ann. Chim. Phys. 28, 433 (1903).

[b28] Juška G., Arlauskas K., Stuchlik J. & Österbacka R. Non-Langevin bimolecular recombination in low-mobility materials. J. Non-Cryst. Solids 352, 1167–1171 (2006).

[b29] Facchetti A. Polymer donor-polymer acceptor (all polymer) solar cells. Mater. Today 16, 123–132 (2013).

[b30] Roland S. . Fullerene-free polymer solar cells with highly reduced bimolecular recombination and field-independent charge carrier generation. J. Phys. Chem. Lett. 5, 2815–2822 (2014). 10.1021/jz501506z 26278084

[b31] Zalar P., Kuik M., Ran N. A., Love J. A. & Nguyen T-Q. Effects of processing conditions on the recombination reduction in small molecule bulk heterojunction solar cells. Adv. Energy Mater. 4, 1400438 (2014).

[b32] Servaites J. D., Yeganeh S., Marks T. J. & Ratner M. A. Efficiency enhancement in organic photovoltaic cells: consequences of optimizing series resistance. Adv. Func. Mater. 20, 97–104 (2010).

[b33] Blom P. W. M., Mihailetchi V. D., Koster L. J. A. & Markov D. E. Device physics of polymer:fullerene bulk heterojunction solar cells. Adv. Mater. 19, 1551–1566 (2007).

[b34] Gevaerts V. S., Furlan A., Wienk M. M., Turbiez M. & Janssen R. A. J. Solution processed polymer tandem solar cell using efficient small and wide bandgap polymer:fullerene blends. Adv. Mater. 24, 2130–2134 (2012). 22438114 10.1002/adma.201104939

[b35] Ouhib F. . Photovoltaic cells based on polythiophenes carrying lateral phenyl groups. Thin Solid Films 516, 7199–7204 (2008).

[b36] Pei Q., Järvinen H., Österholm J. E., Inganäs O. & Laakso J. Poly[3-(4-octylphenyl)thiophene], a new processable conducting polymer. Macromolecules 25, 4297–4301 (1992).

[b37] Yan H. . A high-mobility electron-transporting polymer for printed transistors. Nature 457, 679–686 (2009). 19158674 10.1038/nature07727

[b38] Chen Z., Zheng Y., Yan H. & Facchetti A. Naphthalenedicarboximide vs perylenedicarboximide-based copolymers. Synthesis and semiconducting properties in bottom-gate n-channel organic transistors. J. Am. Chem. Soc. 131, 8–9 (2009). 19090656 10.1021/ja805407g

[b39] Kotlarski J. D., Blom P. W. M., Koster L. J. A., Lenes M. & Sloof L. H. Combined optical and electrical modeling of polymer:fullerene bulk heterojunction solar cells. J. Appl. Phys. 103, 084502 (2008).

[b40] Mihailetchi V. D., Blom P. W. M., Hummelen J. C. & Rispens M. T. Cathode dependence of the open-circuit voltage of polymer:fullerene bulk heterojunction solar cells. J. Appl. Phys. 94, 6849–6854 (2003).

[b41] Wetzelaer G. A. H., Van der Kaap N. J., Koster L. J. A. & Blom P. W. M. Quantifying bimolecular recombination in organic solar cells in steady state. Adv. Energy Mater. 3, 1130–1134 (2013).

[b42] Mihailetchi V. D., Xie H., de Boer B., Koster L. J. A. & Blom P. W. M. Charge transport and photocurrent generation in poly(3-hexylthiopene):methanofullerene bulk-heterojunction solar cells. Adv. Funct. Mater. 16, 699–708 (2006).

[b43] Lu L. & Yu L. Understanding low bandgap polymer PTB7 and optimizing polymer solar cells based on it. Adv. Mater. 26, 4413–4430 (2014). 24677495 10.1002/adma.201400384

[b44] Foertig A. . Nongeminate and geminate recombination in PTB7:PCBM solar cells. Adv. Funct. Mater. 24, 1306–1311 (2014).

[b45] Mingebach M., Walter S., Dyakonov V. & Deibel C. Direct and charge transfer state mediated photogeneration in polymer-fullerene bulk heterojunction solar cells. Appl. Phys. Lett. 100, 193302 (2012).

[b46] Vandewal K. . Efficient charge generation by relaxed charge-transfer states at organic interfaces. Nat. Mater. 13, 63–68 (2014). 24240240 10.1038/nmat3807

[b47] Tang Z. . Improving cathodes with a polymer interlayer in reversed organic solar cells. Adv. Energy Mater. 4, 1400643 (2014).

[b48] Mihailetchi V. D., Wildeman J. & Blom P. W. M. Space-charge limited photocurrent. Phys. Rev. Lett. 94, 126602 (2005). 15903944 10.1103/PhysRevLett.94.126602

