# Supplementary material for: Competition between recombination and extraction of free charges determines the fill factor of organic solar cells
Source: Nat Commun. 2015 May 7;6:7083. doi: 10.1038/ncomms8083 (PMC4432638; doi:10.1038/ncomms8083)
Supplement: Supplementary Information — Supplementary Figures 1-7, Supplementary Note 1 and Supplementary References [file ncomms8083-s1.pdf]

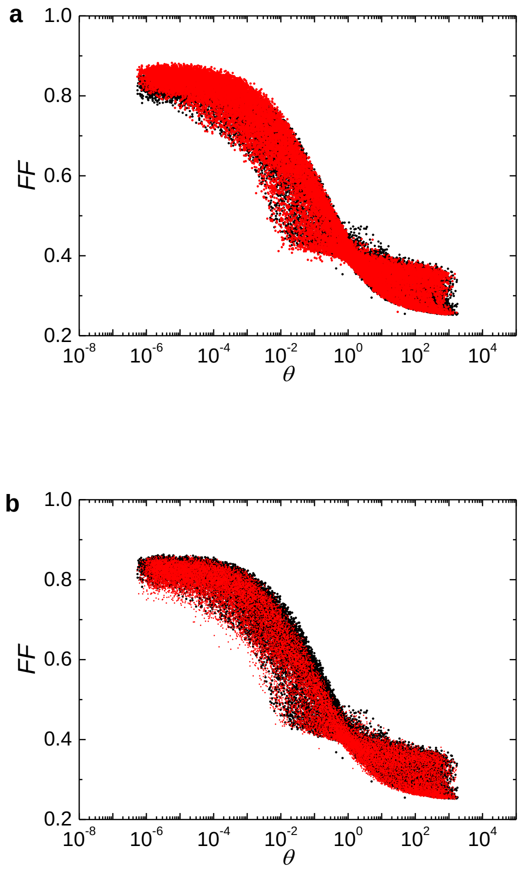

Supplementary Figure 1 (a) The effect of finite surface recombination (red symbols, $S_{n,p}$ = 10^{-4} m s^{-1}) and infinite surface recombination (black symbols). **(b)** Resulting fill-factors for $N_{cv} = 2.5 \times 10^{25}$ m^{-3} (black) and $N_{cv} = 3 \times 10^{26}$ m^{-3} (red).

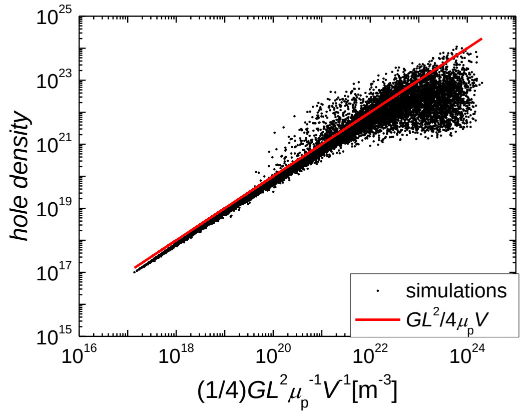

Supplementary Figure 2. Simulated density of holes at the cathode-side of the active layer as a function of $GL^2/\mu_p V_{int}$. The solid lines is calculated with the equation $p_{av} = \dfrac{GL^2}{4\mu_p V_{int}}$.

Supplementary Figure 3:

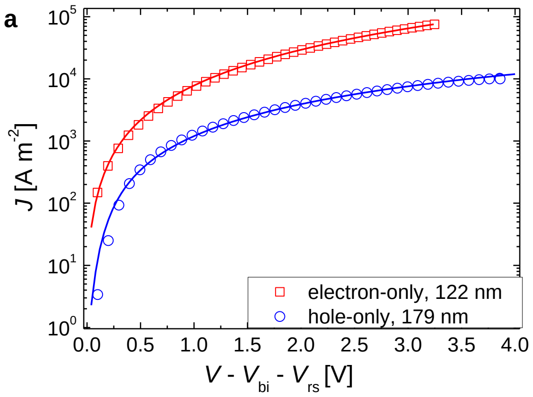

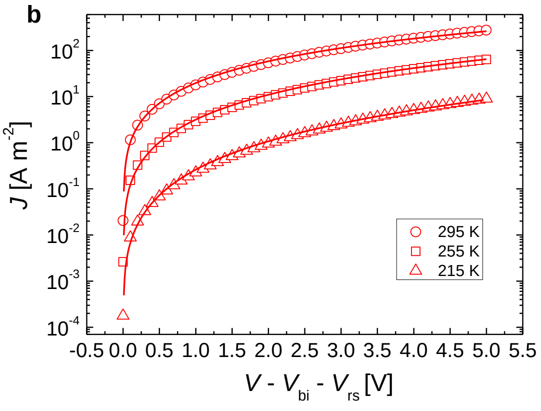

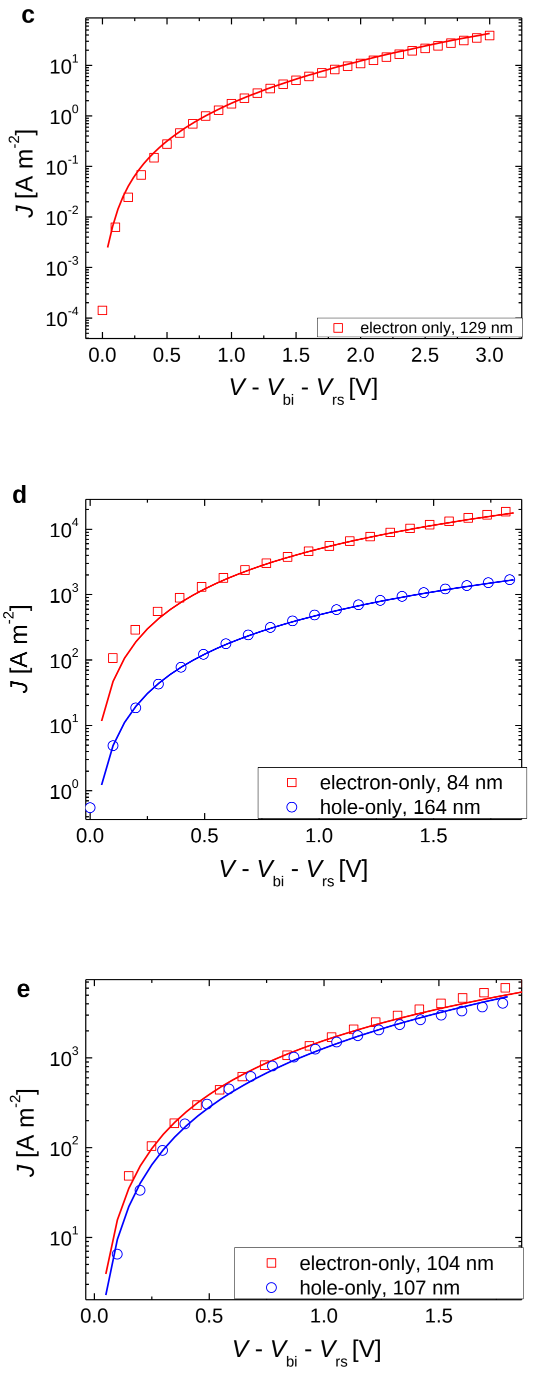
c
electron only, 129 nm
J [A m^-2]
V - V_bi - V_rs [V]
d
electron-only, 84 nm
hole-only, 164 nm
J [A m^-2]
V - V_bi - V_rs [V]
e
electron-only, 104 nm
hole-only, 107 nm
J [A m^-2]
V - V_bi - V_rs [V]

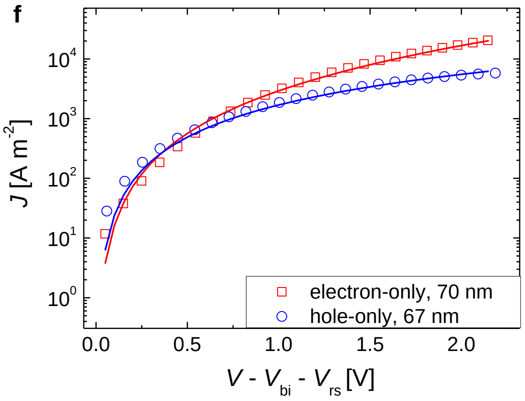

Supplementary Figure 3: *J-V* curves of PDPP5T:[70]PCBM and PTB7:[70]PCBM hole-only and electron-only devices. **(a)** PDPP5T:[70]PCBM 1:2 wt. ratio; **(b)** PDPP5T:[70]PCBM 1:1 wt. ratio; **(c)** PDPP5T:[70]PCBM 2:1 wt. ratio; **(d)** PTB7:[70]PCBM 1:1.5 wt. ratio from CB solution; **(e)** PTB7:[70]PCBM 1:1.5 wt. ratio from oDCB solution; **(f)** PTB7:[70]PCBM 1:1.5 wt. ratio from CB/DIO solution. Symbols represent experimental data, corrected for the series resistance of the substrates (10 Ω/square for the electron-only devices, 14 Ω/square for the hole-only devices) and for the built-in voltage. Solid lines represent fit of the experimental data, performed for space charge limited current with a Poole-Frenkel dependency for the mobility on the electric field[1]. The mobilities of holes and electrons are thus extracted as fit parameters from the *J-V* curves. For PDPP5T:[70]PCBM blends, the hole only was not measured for all the compositions; when the hole-only device was not fabricated, we got the hole mobility from a previous publication[2].

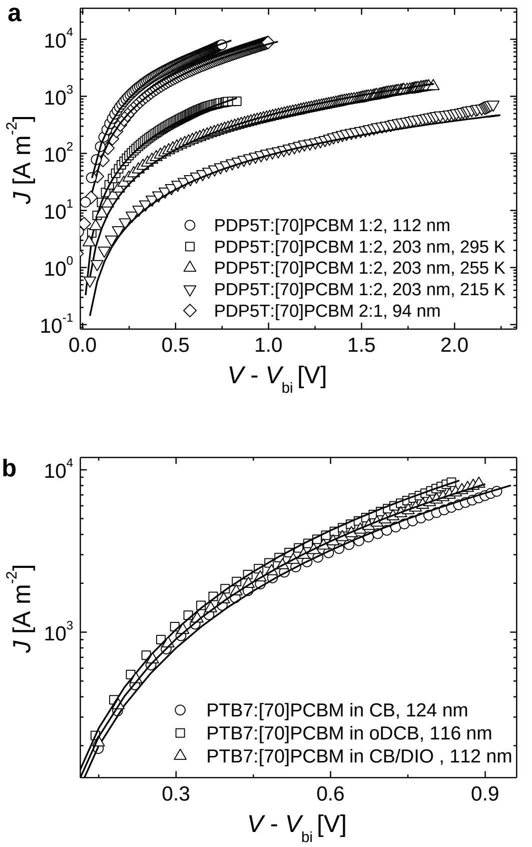

Supplementary Figure 4: *J*-*V* curves in dark for PDPP5T:[70]PCBM **(a)** and PTB7:[70]PCBM **(b)** solar cells. Symbols represent experimental data, solid lines represent fit of the experimental data.

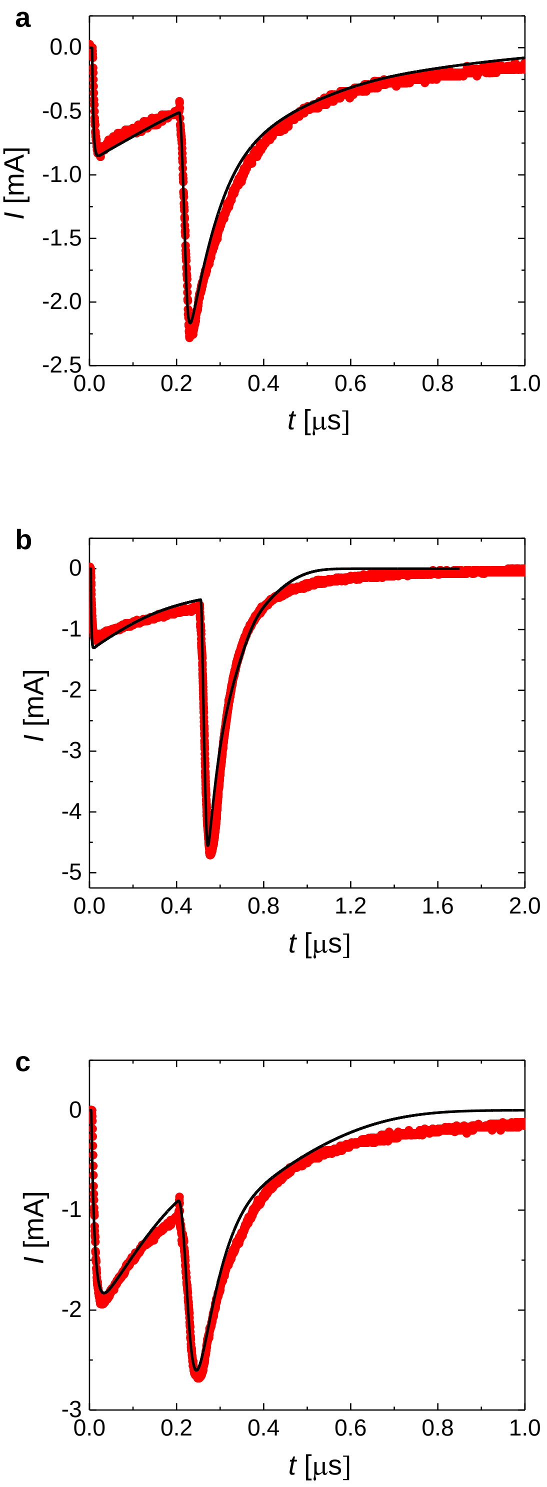
a
b
c
I [mA]
t [μs]
0.0
-0.5
-1.0
-1.5
-2.0
-2.5
0.2
0.4
0.6
0.8
1.0
0
-1
-2
-3
-4
-5
1.2
1.6
2.0

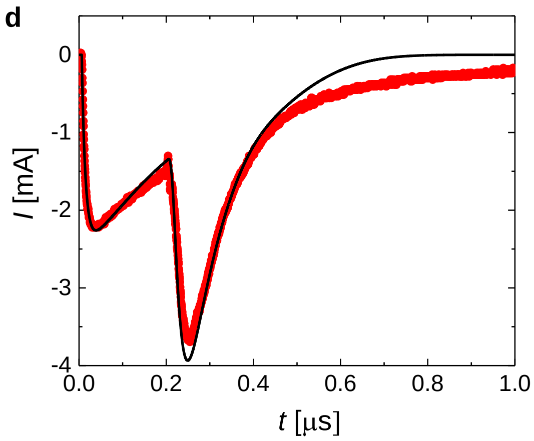

Supplementary Figure 5: TDCF transients of the polymer:polymer devices: P3HT:P(NDI2OD-T2) **(a)**, P3HT:P(PDI2OD-T2) **(b)**, POPT:P(NDI2OD-T2) **(c)**, POPT:P(PDI2OD-T2) **(d)**. The symbols represent experimental data, the solid lines represent fits with a numerical transient drift-diffusion simulation[3-5].

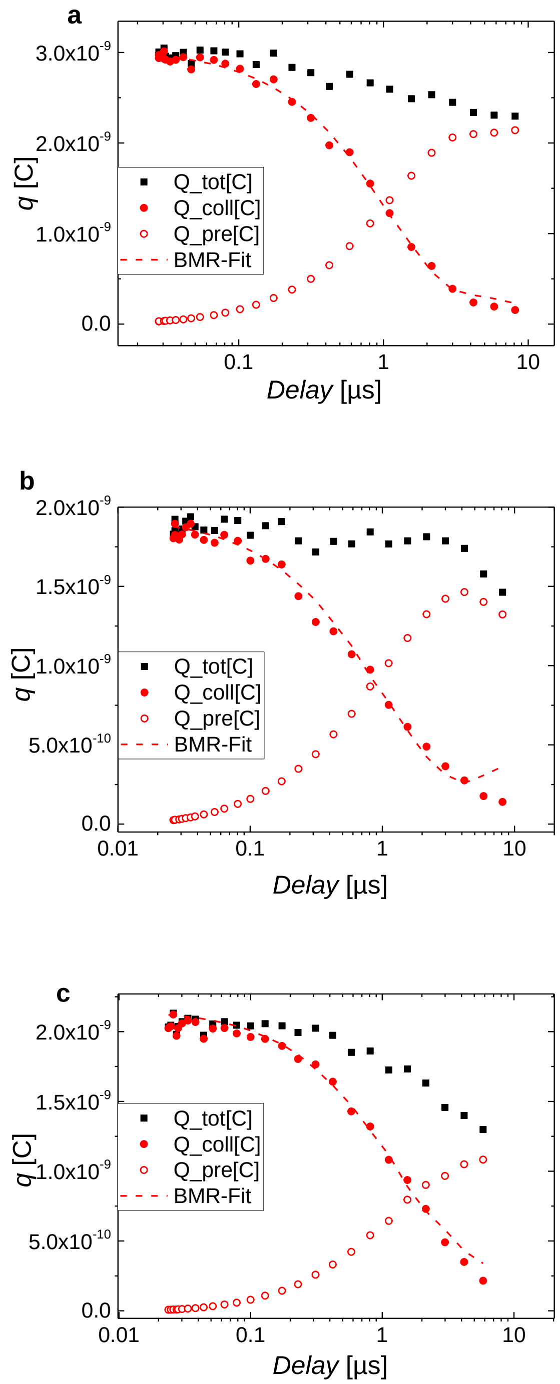

Supplementary Figure 6: Delay dependent total (Q_{tot}, black squares), collection (Q_{coll}, red full circles) and pre-charge (Q_{pre}, red empty circles) for P3HT:P(PDI2OD-T2) (a), POPT:P(NDI2OD-T2) (b) and POPT:P(PDI2OD-T2) (c). A pre-bias close to the maximum power point of each solar cell has been applied. The bimolecular recombination coefficients γ has been extracted from the fit of Q_{coll} with the equation[2]

$$Q_{coll}(t_D+\Delta t)=Q_{coll}(t_D)-[Q_{pre}(t_D+\Delta t)-Q_{pre}(t_D)]-\frac{\gamma}{eAd}[Q_{coll}^2(t_D)+2Q_{coll}(t_D)Q_{bg}]\Delta t \qquad (1)$$

Here, A and d are the device area and thickness, Q_{bg} is the background charge that arises due to dark injection from the contacts at forward bias.

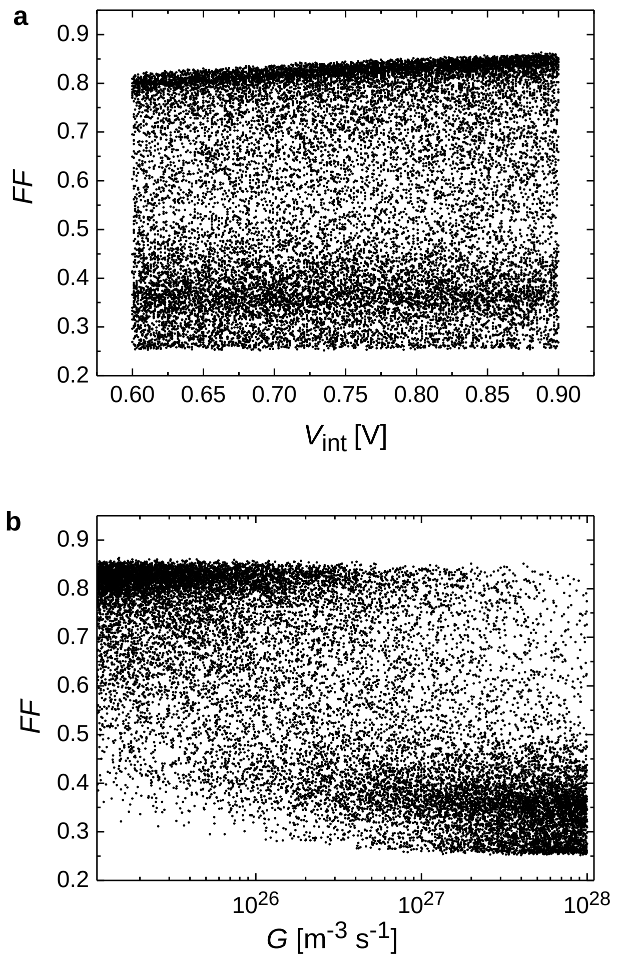

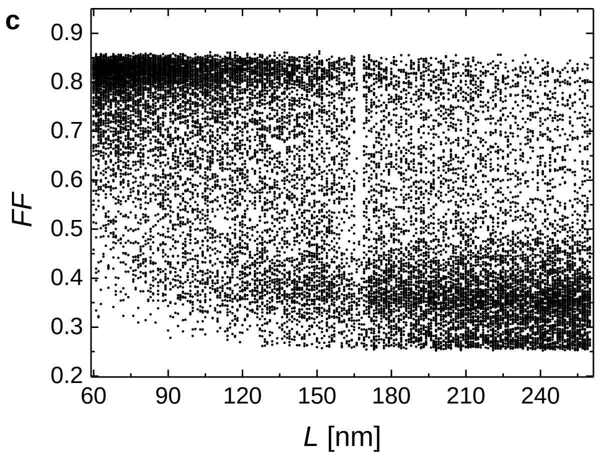

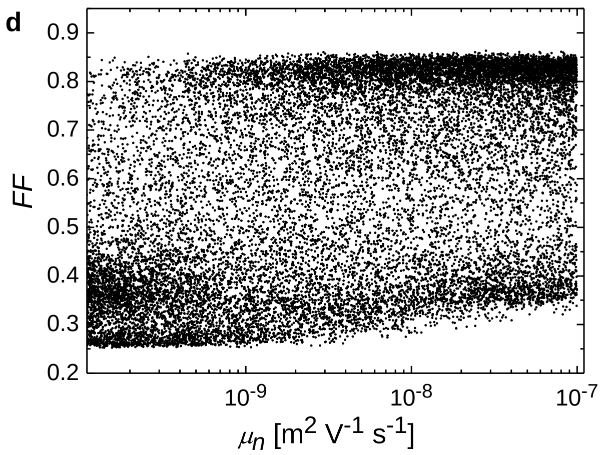

Supplementary Figure 7: *FF* vs V_{int} (**a**), *G* (**b**), *L* (**c**) and μ_{n} (**d**) for the simulated data.

Supplementary Note 1 - Carrier densities at the contacts

In the case of thermal equilibrium at the contacts, it is assumed that the electron and hole density at the cathode (x=0) are equal to

$$n(0) = N_{\mathrm{cv}} \qquad (2)$$

$$p(0) = N_{\mathrm{cv}} \exp\left(-\frac{E_{\mathrm{g}}^{\mathrm{eff}}}{kT}\right), \qquad (3)$$

where N_{cv} is the density of available states [REFKoster2005], and $E_{\mathrm{g}}^{\mathrm{eff}}$ is $LUMO_A$ - $HOMO_D$. At the anode the densities are given by

$$n(L) = N_{\mathrm{cv}} \exp\left(-\frac{E_{\mathrm{g}}^{\mathrm{eff}}}{kT}\right) \qquad (4)$$

$$p(L) = N_{\mathrm{cv}} \qquad (5)$$

In Ref. 6 it is assumed that $N_{\mathrm{cv}} = 2.5 \times 10^{25}$ m^{-3}. If there is 1 hop site per 1.5 nm, then $N_{\mathrm{cv}} = 3 \times 10^{26}$ m^{-3}.

In the case of near-zero surface recombination, the boundary conditions on *p(0)* and *n(L)* are replaced by the requirement that the hole, respectively, electron current densities are zero at cathode and anode. This is done by assuming

$$J_{\mathrm{n(p)}} = qS_{\mathrm{np}}(n(p) - n(p)_{\mathrm{eq}}) \qquad (6)$$

where S_{np} is the surface recombination velocity, where $n(p)_{\mathrm{eq}}$ is the equilibrium electron (hole) density[5]. Near-zero recombination velocity is achieved by setting $S_{\mathrm{n,p}} = 10^{-4}$ m/s.

Supplementary References

1. Frenkel, J. On pre-breakdown phenomena in insulators and electronic semiconductors. *Phys. Rev.* **54,** 647–648 (1938).

2. Bartesaghi, D., Turbiez, M., Koster, L. J. A. Charge transport and recombination in PDPP5T:[70]PCBM organic solar cells: the influence of morphology. *Org. Elect.* **15,** 3191–3202 (2014).

3. Albrecht, S., Janietz, S., Schindler, W., Frisch, J., Kurpiers, J., Kniepert, J., Inal, S., Pingel, P., Fostiropoulos, K., Koch, N. & Neher, D. Fluorinated copolymer PCPDTBT with enhanced open-circuit and reduced recombination for highly efficient polymer solar cells. *J. Am. Chem. Soc.* **134,** 14932–14944 (2012).

4. Roland, S., Schubert, M., Collins, B. A., Kurpiers, J., Chen, Z., Facchetti, A., Ade, H. & Neher, D. Fullerene-free polymer solar cells with highly reduced bimolecular recombination and field-independent charge carrier generation. *J. Phys. Chem. Lett.* **5,** 2815–2822 (2014).

5. Kniepert, J., Lange, I., van der Kaap, N. J., Koster, L. J. A. & Neher, D. A conclusive view on charge generation, recombination, and extraction in as-prepared and annealed P3HT:PCBM blends: combined experimental and simulation work. *Adv. Energy Mater.* **4,** 1301401 (2014).

6. Koster, L. J. A., Smits, E. C. P., Mihailetchi, V. D. & Blom, P. W. M. Device model for the operation of polymer/fullerene bulk heterojunction solar cells. *Phys. Rev. B* **72,** 085205 (2005).
